# Medical College Notes

**Published:** 1890-09

**Authors:** 


### Medical College Note.

Chicago Policlinic.—The Faculty of this institution have made the following appointments:

Dr. G. Fütterer (late chief-assistant to Professor Rindflesh, of Würzburg), Drs. F. C. Hotz and E. Fletcher Ingalls, Professors of Internal Medicine, Ophthalmology, and Laryngology, respectively; also Drs. Chas. F. Stillman, P. S. Hayes and J. M. Patton, Associate Professors of Orthopedic Surgery, Electro-Therapeutics, and Medicine, respectively.

